# Understanding the experiences of anger in the onset and progression of psoriasis: A thematic analysis

**DOI:** 10.1002/ski2.111

**Published:** 2022-03-17

**Authors:** Olivia Hughes, Rachael Hunter

**Affiliations:** ^1^ School of Psychology Cardiff University Cardiff UK; ^2^ Department of Psychology Swansea University Swansea UK

## Abstract

Psoriasis is a chronic inflammatory skin condition, which can be affected by stress. Living with psoriasis can trigger negative emotions, which may influence quality of life. The present study explored the lived experiences of people with psoriasis with attention to the potential role of anger in the onset and progression of the chronic skin condition. Semi‐structured qualitative interviews were conducted with 12 participants (*n* = 5 females, *n* = 7 males) recruited from an advert on a patient charity social media platform. Data were transcribed and analysed using thematic analysis. Four key themes were identified: (1) anger at the self and others, (2) the impact of anger on psoriasis: angry skin, (3) shared experiences of distress and (4) moving past anger to affirmation. Findings suggest that anger can have a perceived impact on psoriasis through contributing to sensory symptoms and unhelpful coping cycles, and points to a need for enhanced treatment with more psychological support. The findings also highlight the continued stigma which exists for those living with visible skin conditions and how this may sustain anger for those individuals. Future research could usefully focus on developing targeted psychosocial interventions to promote healthy emotional coping.

## Graphical abstract

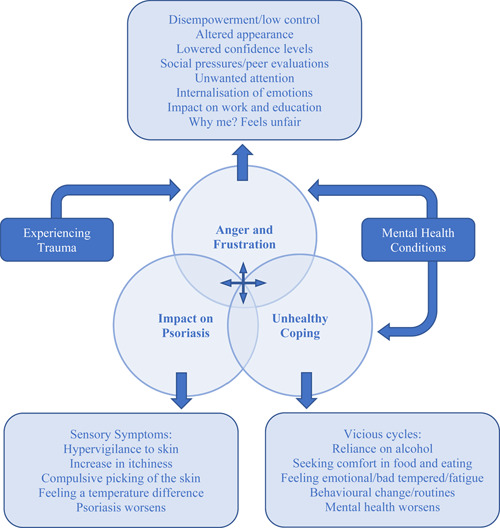

1

**What is already known about this topic?**

- Psoriasis is a chronic inflammatory skin condition that has been associated with emotional and psychological distress in some people.
- Stress has been found to impact on psoriasis, and has been linked to flare‐ups.
- It has been suggested that anger could occur as an outcome of living with a visible skin condition.

**What does this study add?**

- This study is the first, to our knowledge, to use qualitative methods to explore the lived experiences of anger and the role of negative emotion in the onset and progression of psoriasis.
- Findings suggest that anger may play a contributory role in the experience of psoriasis for some patients.
- The potential contributory role of experiencing trauma was highlighted, with psoriasis in some cases, acting as a secondary trauma.

**What are the clinical implications of this work?**

- To optimize psoriasis treatments, patient emotional and psychological wellbeing must be considered, to facilitate healthy coping.
- There may be value in routinely addressing patient experiences of humiliation and anger, as this is not normally assessed with mainstream mood measures.

## 2 INTRODUCTION

Psoriasis is a chronic inflammatory skin condition arising from the excess production of cells in the upper epidermis, causing raised or thickened patches of skin on the body.^1^ There are several types of psoriasis which can all fluctuate, and differ in presentation, including plaque (large scaly patches), guttate (small red patches), pustular (with pustules) and erythrodermic (covering most of the body) which can all occur alongside psoriatic arthritis.^1^, ^2^

Although psoriasis is an autoimmune condition, it is multifactorial in expression^3^, ^4^ as emotional life events can influence onset and clinical course. Research has focussed on the role of emotions^5^, ^6^ and stressful or traumatic life events triggering psoriasis^7^, ^8^, ^9^; however, studies have reported mixed findings.^10^, ^11^, ^12^ As such, questions remain about the role of emotions and experiences, their relationship with symptom severity, and the mechanisms of which trauma may interact with inflammatory markers.^13^ The roles of emotion and stress in psoriasis are complicated by visibility, with Western society valuing beauty and myths existing regarding broken skin.^1^, ^14^ Having noticeable changes to the skin could cause unwanted attention, remove an individual's control over when they disclose their condition or prompt people to dress differently, attracting more attention.^14^ This might explain why skin conditions have been associated with depression,^14^, ^15^, ^16^, ^17^ low self‐esteem and maladaptive self‐beliefs or appearance anxiety.^18^, ^19^, ^20^ In an attempt to avoid negative evaluations and humiliation, people with psoriasis may anticipate rejection, withdraw socially^21^ or experience shame.^13^, ^22^

Anger has been reported in people with skin conditions^23^ including alopecia,^4^ urticaria^24^ and psoriasis^4^, ^25^, ^26^ from their uncontrollable nature, or as an outcome of poor mental health. Experiencing anger could impede immune function in a similar way to the stress response^6^ and exacerbate skin severity or disease duration.^5^, ^22^ For example, an ‘itch–scratch cycle’ could be triggered by the psychological burden of psoriasis and the internalization of anger, resulting in a behavioural response of scratching.^27^

Focussing on the psychological impact of skin conditions has long been seen as necessary^28^ if patient treatments are to be optimized, with calls growing for interventions to be more psychologically informed.^29^, ^30^ However, the All‐Party Parliamentary Group on Skin^31^ reported that 98% of people surveyed felt psychologically negatively impacted by their skin condition, but only 18% had received support. Despite the increasing interest in psychosocial aspects of autoimmune conditions, there is a scarcity of research examining anger associated with psoriasis. The purpose of this study was to address this gap by exploring the onset and progression of psoriasis with attention to the role of anger.^32^ To our knowledge, there has been no study to date which has qualitatively investigated anger related to psoriasis. Such an approach has a growing body of literature examining a range of health conditions^33^, ^34^ including burns,^35^ and visible difference.^36^, ^37^

## 3 MATERIALS AND METHODS

### 3.1 Participants

Using purposive sampling, 12 participants were recruited in collaboration with the Psoriasis Association UK for semi‐structured interview. An advertisement on social media was used to inform that for inclusion, participants had to be (a) aged over 18 years, and (b) be diagnosed with psoriasis. Participants (*n* = 5 females, *n* = 7 males) were aged 26–74 years (mean age = 45.16) and were all British. The age of diagnosis of psoriasis ranged from 5 to 46 years (mean age = 19.42), and presentations ranged from guttate, plaque, to severe erythrodermic, and psoriatic arthritis (Table 1).

**TABLE 1:** Participant information

Pt no.	Age range in years	Gender	Psoriasis type
1	30–40	Female	Guttate/plaque
2	20–30	Male	Plaque
3	60–70	Male	Plaque/psoriatic arthritis
4	30–40	Female	Guttate/plaque
5	60–70	Male	Plaque
6	40–50	Male	Plaque
7	70–80	Male	Plaque/guttate
8	50–60	Male	Plaque/erythrodermic/psoriatic arthritis
9	20–30	Female	Guttate/plaque/psoriatic arthritis
10	40–50	Male	Plaque/psoriatic arthritis
11	20–30	Female	Plaque
12	40–50	Female	Plaque

### 3.2 Procedure

Ethical approval was granted by University Departmental Ethics (ref: 3773). Participants responded to the online advertisement and were given a choice of interview to promote empowerment^38^; face to face, Skype or telephone, which facilitated choice over visibility.^37^ Drawing on previous qualitative research,^39^ a semi‐structured interview schedule was developed, allowing participants to discuss topics they felt relevant (Table 2). The interviews lasted approximately 40 min and were audio‐recorded.

**TABLE 2:** Example interview questions

Question
• To start with, could you tell me about when were you first diagnosed with psoriasis?
• How does having psoriasis affect you?
• Does your psoriasis fluctuate?
• Do you find your psoriasis is affected by emotions? What about anger?

Braun and Clarke^40^ report that meaning in qualitative research is constructed from subjective interpretations, so data collection cannot be predicted with an estimate of required interviews, or with data saturation alone.^40^, ^41^, ^42^, ^43^ Therefore, based on the study aims and the strength of interview dialogue, recruitment was continuously evaluated until there was consensus of the data being extensive in information power.^40^, ^44^

### 3.3 Data analysis

Data were analysed with thematic analysis.^40^, ^41^, ^45^ Interviews were transcribed verbatim, and participants were assigned numbers to protect identities. Verbal comments in the form of quotes were used to preliminary code transcripts, extending to full coding across the dataset, and themes were developed based on shared meanings.^40^, ^41^ To ensure a valid interpretation of the phenomenon, researchers assessed information power^44^ by reviewing data to check themes were relevant to the research aim.^40^, ^41^, ^44^

## 4 RESULTS

Thematic analysis revealed four themes and seven subthemes (Table 3).

**TABLE 3:** Main themes and subthemes

Themes	Subthemes
1. Anger at the self, and others	1.1. Disempowerment
	1.2. Social evaluations
2. The impact of anger on psoriasis: angry skin	2.1. Sensory symptoms and emotion
	2.2. Precipitating vicious cycles
3. Shared experiences of distress	3.1. Comorbid mental health conditions
	3.2. Experiencing trauma
4. Moving past anger to affirmation	4.1. Protective factors buffering the negative impact of psoriasis

### 4.1 Theme 1: Anger at the self and others

Feeling angry became an organizing principle, with anger aimed at psoriasis itself, and the impact it had on daily life.

#### 4.1.1 Disempowerment

Most participants felt that they were not in control of their psoriasis, which precipitated disempowerment:

It's anger at the condition…no matter what I do, I cannot get a handle of it… it's anger that it’s not within your control. (Participant 11).

I can't do anything…I'm really really angry about the situation. (Participant 6).

This triggered anger which affected mood, and included adjustment to altered appearance/changing self‐perception:

Any feelings of anger I cry…I'm irritated and it's because I'm feeling so out of control in my own body. (Participant 1).

There's been a lot of tears of frustration… I'm sick of the itching and my skin bleeding and cracking and looking like it does…it's a grieving process for the life you used to have. (Participant 9).

Participants described ‘a rage towards yourself’ (participant 2) for their appearance and not achieving their potential, and attributed this ‘loss’ to psoriasis:

I get angry with myself…because of the frustration of it…and of not being able to do stuff that I really wanted. (Participant 8).

I feel angry when I look at my skin…‘why me?’…it makes me frustrated…that's where the anger comes from. (Participant 11).

Not feeling one‐hundred percent confident in…my own skin. (Participant 2).

Participants described being self‐conscious as they could not control their personal presentation, and often engaged in checking behaviours to see if their skin was shedding:

I'd like to feel one‐hundred percent confident…I just get really angry that I have to be double‐checking myself. (Participant 12).

Feeling disempowered extended socio‐economically with reduced performance and missed opportunities:

I've seen all my friends do well…got nice cars…nice houses…and I'm in my fifties and live with my parents…I've missed out…that's been the biggest frustration…I've not fulfilled my potential. (Participant 8).

That definitely did affect my university performance…and my job performance. (Participant 11).

#### 4.1.2 Social evaluations

Negative comments from other people were distressing, abusive, and an ongoing source of anger. In some cases, this led to participants becoming ‘reclusive at times’ (participant 2):

I have been angry, when the guy called us a leper colony…if I could have caught up with him I would have smashed his face in. (Participant 8).

What causes the most frustration…other people's perceptions…it still gets really really frustrating. (Participant 12).

Someone said to me ‘you're very unlucky, you're bald and you have dandruff’…you get self‐conscious. (Participant 3).

I had a bandage on my head, they called me cyclops. (Participant 7).

Notably, anger extended to participants evaluating themselves compared to others, for example, not being able to wear the same clothes as friends:

I get very frustrated…we're going away with my friends soon…they're all talking about outfits and I'm getting really annoyed because I'm just going to be in trousers. (Participant 4).

One participant felt that having psoriasis since childhood had impacted on psychosexual development and contributed to negative self‐beliefs:

I'm in my fifties…never been married, haven't got a girlfriend so it's affected me…when you're in your late teens and early twenties and your mates are getting girlfriends…that passed me by… because I was self‐conscious of it…my skin being bad, and thinking ‘who would want me?’…’I'm a mess’. (Participant 8).

### 4.2 Theme 2: The Impact of Anger on Psoriasis: Angry Skin

Participants described how they felt their feelings manifested physically, highlighting the bidirectionality of emotion/body symptoms.

#### 4.2.1 Sensory symptoms and emotion

Sensory symptoms were described when feeling emotional which was metaphorical for one participant: ‘It's crawling on you like bugs’ (participant 1), while others described exacerbation with sensory vigilance: ‘If I'm feeling down…I tend to feel my psoriasis more’ (participant 10).

Some mentioned a temperature change in the skin when angry, and in one case, the ‘whole body burned’ (participant 6):

If you do get angry…it's really quick to see the difference in my skin…the psoriasis I have will become redder, more‐scaly…my skin feels hotter. (Participant 11).

If you touch it, you can feel a heat in the psoriasis…there's a temperature difference. (Participant 6).

#### 4.2.2 Precipitating vicious cycles

Anger appeared to maintain a vicious cycle of emotions/unhealthy coping. It appeared that anger not only worsened symptoms, but feelings of anger towards symptoms were exacerbated:

It just spread so quickly…I was getting upset and stuff about it and I'm sure that accelerated it. (Participant 9).

There was a vicious circle that I'd get tired and I'd get bad tempered, I'd get psoriasis, I'd get more‐bad tempered, I'd get more tired. (Participant 3).

I got stuck in a cycle of frustration, depression, anger, anxiety, a lot of anxiety from stress and…the heat of anger. (Participant 6).

These emotions related to psoriasis led participants to respond in a range of ways, some of which were maladaptive and maintained vicious cycles:

I was self‐medicating with alcohol…the worse it got, the worse I drank, the worse it got. (Participant 8).

I'll get really angry…I'm angry cause I'm stressed, and then my skin will flare, and then if I'm stressed it's cause I'm pissed off and angry, and then my skin will flare…then it's a vicious circle…you feel like shit so you eat, you feel better for a little bit, and then you feel like shit because you've eaten…it's a downward spiral. (Participant 12).

### 4.3 Theme 3: Shared experiences of distress

Participants discussed similar experiences of mental health conditions, distress, trauma, and the mediating role of confidence.

#### 4.3.1 Comorbid mental health conditions

Comorbid mental health conditions exacerbated the emotional impact of psoriasis from determining behavioural responses, including anxiety related to appearance and fear of judgement:

It was anxiety of people asking me about it and being different. (Participant 2).

I spent an hour in the bathroom crying before I went to the pool because… you could see the psoriasis…I was like so paranoid. (Participant 11).

Mental health presentations were attributed to psoriasis, and importantly, both resulted in and contributed to emotional lability:

I have reoccurring manic depression…and I've got borderline personality disorder…which means I'm overly‐emotional. (Participant 12).

I don't like being out of control…I have OCD. (Participant 1).

Some participants described a compulsion to pick their skin which was triggered by frustration and drove self‐directed emotion:

I pick, when I'm anxious, when I'm frustrated…which makes it worse and then I get really pissed off with myself…it's sore…it's bleeding. (Participant 12).

I've been…through a big phase of picking…then I got hair loss…I'm not pulling my hair out I'm pulling my psoriasis out. (Participant 1).

#### 4.3.2 Experiencing trauma

Anger was often precipitated by traumatic experiences, notably, with historical trauma being linked to the onset of psoriasis. This anger contributed to psychological distress and influenced affect through rumination:

When I was 12, I was diagnosed with psoriasis…it was put down to the fact that my mother died when I was 6. (Participant 7).

It stems from…a rape… that's what triggered my psoriasis…if I had spoken to somebody about it back then… I might not have psoriasis…I was an angry little person. (Participant 4).

I'm really really angry…I was abused years ago and it keeps coming into my head. (Participant 6).

I think with the miscarriage and my psoriasis being so bad I'm not calm. (Participant 1).

### 4.4 Theme 4: Moving past anger to affirmation

Participants described factors which had lessened their anger and helped them manage their psoriasis.

#### 4.4.1 Protective factors buffering the negative impact of psoriasis

Romantic relationships helped participants feel more ‘assured in yourself’ (participant two), along with friends and family:

My husband makes me feel good. (Participant 12).

My wife…she's been very supportive. (Participant 7).

I have got some really good friends that are very supportive. (Participant 9).

Connecting with other people with psoriasis helped ‘to know that other people out there are going through similar things and are coping well’ (participant 11). One participant found ‘peace in reading the Bible’ (participant 7). In addition, age promoted acceptance, from growing older with the condition, and age at onset:

I've got more blasé…I will now wear short‐sleeved shirts even though I've got psoriasis on my elbows…there was a time when I wouldn't have. (Participant 7).

I've been very lucky in so much that I didn't have it as a child when I suspect it would have affected me much more. (Participant 5).

I got it when I wasn't as vulnerable….the thought of being a teenager with psoriasis is pretty chilling. (Participant 3).

Some participants described gaining experience in managing psoriasis, which made them ‘less angry about it now’. (Participant 8) as they had learnt to treat it:

It's experience… I know that a flare‐up is coming so I take precautions to make sure that it’s not as bad. (Participant 8).

I've had it that long I've got used to it. (Participant 10).

However, one participant had received specialized support for their psoriasis, which was a ‘life‐changer’ (participant 8):

I'd probably be dead by now if it was not for that intervention…it had got that bad. (Participant 8).

## 5 DISCUSSION

**FIGURE 1:**
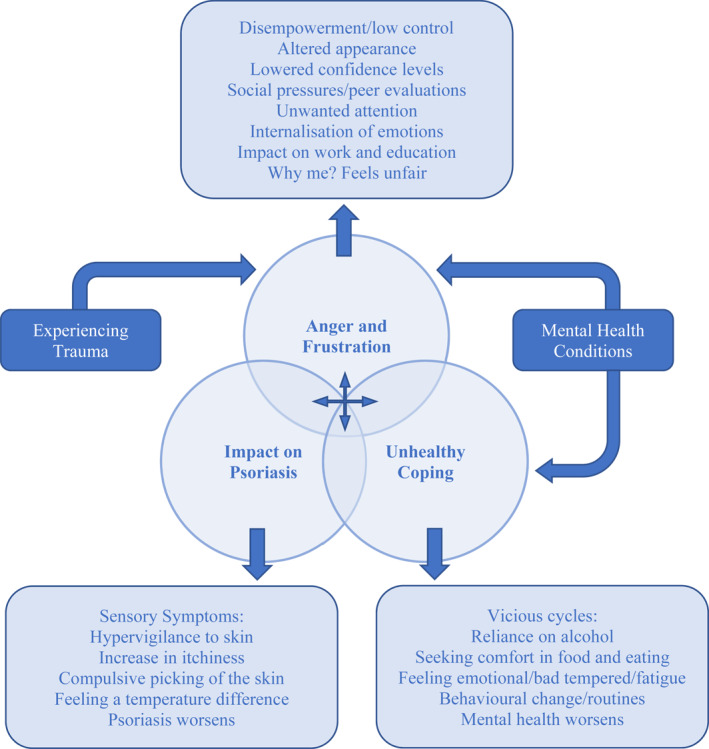
A model representation of participants experiences of anger and frustration in psoriasis, as reported in this study.

This study explored the experiences of people with psoriasis, with attention to the role of anger. Our findings support previous research,^4^, ^5^, ^22^, ^25^, ^26^ shed light on the perpetuating factors for anger, and to our knowledge, is the first study to qualitatively investigate anger and psoriasis. The themes identified demonstrate that anger was experienced from psoriasis itself, feeling out of control, and life circumstances^46^ which all perpetuated anger^22^ and created vicious cycles of emotion/increased symptoms. As illustrated in Figure 1, participants described how anger emerged and was exacerbated by anxiety, low self‐esteem, and lack of confidence.^4^, ^23^ Self‐directed and internalized frustrations were expressed at having to alter personal presentation and adjusting to changing self‐perceptions. A twofold process of social evaluations was outlined; firstly, individuals were negatively evaluated by other people,^14^ triggering social avoidance; and secondly, evaluated themselves compared to others, and felt frustrated at covering their skin and not achieving their ‘potential’. For some, this sense of underachieving resulted from a disruption to psychosexual maturation in adolescence lasting into early adulthood with negative beliefs about self/desirability being maintained in later life.^19^, ^20^

The complexities of backgrounds influenced anger, with the trauma reported in this study being similarly found in psoriasis^7^, ^10^, ^11^ and other autoimmune conditions.^47^ Participants perceived that anger caused by trauma had negatively impacted their health and contributed to *both* the onset and progression of psoriasis, suggesting that the challenges of living with psoriasis could serve as a secondary trauma.

Findings support the stress–disease cycle^48^ with the stress of psoriasis contributing to disease itself and causing further stress as an outcome. For example, the efforts of participants to self‐manage their stress from psoriasis resulted in maladaptive coping, such as alcoholism^49^ and overeating. This highlights potential targets to support patients in developing adaptive coping strategies, as enhancing self‐efficacy could indirectly improve physical symptoms through stress reduction. Similarly, co‐morbid mental health conditions influenced behaviour and emotion, such as increasing sensory awareness of psoriasis, suggesting a quasi‐sensory hyperawareness.^50^, ^51^ The findings included compulsive picking alongside co‐morbid obsessive‐compulsive disorder and borderline personality disorder, supporting the ‘itch–scratch cycle’^27^ or evidencing psychological symptoms.^52^, ^53^, ^54^ The close, symbolic relationship between anger, heat and redness/inflammation that is commonly metaphorical was reported as a lived, physical sensation, with skin temperature changes when angry. While there is limited research on blood flow in psoriatic plaques, temperature differences could be explained by basal flux/emissivity in psoriatic regions.^55^, ^56^

Despite the challenges, not all individuals experienced severe anger, but instead described the occasional frustration. This was explained by several protective factors, including acceptance^14^ gaining experience, perceived support/connecting with others, romantic relationships^23^, ^36^ spiritual frameworks, and professional intervention. These could be a central focus of future interventions to therapeutically buffer the negative effects of psoriasis and encourage healthy coping.^57^, ^58^

### 5.1 Implications

This study highlights the need for clinicians to consider psychosocial factors in the management of psoriasis, and for further research to improve understandings of the role of emotions to enhance patient wellbeing; physically and psychologically. It may be worthwhile considering experiences of humiliation and anger, as these are not assessed with mainstream mood measures. Future research could also examine the relationship between historical trauma and severity of psoriasis, and the relationship between historical trauma and secondary, appearance or symptom related trauma. Herein may lie targeted psychosocial strategies which may indirectly contribute to the moderation of associated auto‐immune inflammatory responses through enhancing adaptation.^59^, ^60^ There may be opportunities for clinicians to incorporate such interventions practically, or at developmental stages of vulnerability.^61^

### 5.2 Limitations

There were several limitations to this study. Participants were all British (not representing non‐British/BAME patients), complex in backgrounds and self‐selecting (no measure of psoriasis severity). Thus, findings may only reflect the experiences of a subgroup of people who felt motivated to discuss emotions and psoriasis. Future studies could explore these concepts in a wider demographic or employ quantitative methods with standardized psychometric measures. Prospective, longitudinal studies could also be used to observe the evolving experience, and adaptations required for adjusting to psoriasis.

## 6 CONCLUSION

Anger was common for participants, and the causes reflect a complex interplay between developmental stage, symptom severity, social circumstance and trauma. However, the findings suggest anger could arise from adjusting to living with a visible skin condition. While expensive systemic/biologic drugs may offer a quick solution to the physical symptoms of psoriasis, they cannot resolve the underlying emotional burden. Findings from this study suggest that to optimize psoriasis treatments, treatment and management regimens must extend beyond physical symptoms and address contributory psychosocial factors.

## CONFLICT OF INTEREST

The authors report no conflict of interest.

## ETHICS STATEMENT

Ethical approval was granted by University Departmental Ethics (ref: 3773).

## AUTHOR CONTRIBUTIONS

Olivia Hughes: Conceptualization – ideas; formulation or evolution of overarching research goals and aims; data curation – management activities to annotate (produce metadata), scrub data and maintain research data (including software code, where it is necessary for interpreting the data itself) for initial use and later re‐use; formal analysis – application of statistical, mathematical, computational, or other formal techniques to analyse or synthesize study data; investigation – conducting a research and investigation process, specifically performing the experiments, or data/evidence collection; methodology – development or design of methodology; creation of models; project administration – management and coordination responsibility for the research activity planning and execution; resources – provision of study materials, reagents, materials, patients, laboratory samples, animals, instrumentation, computing resources, or other analysis tools; validation – verification, whether as a part of the activity or separate, of the overall replication/reproducibility of results/experiments and other research outputs; visualization – preparation, creation and/or presentation of the published work, specifically visualization/data presentation; writing – original draft – preparation, creation and/or presentation of the published work, specifically writing the initial draft (including substantive translation). Rachael Hunter: Conceptualization – ideas; formulation or evolution of overarching research goals and aims; methodology – development or design of methodology; creation of models; project administration – management and coordination responsibility for the research activity planning and execution; validation – verification, whether as a part of the activity or separate, of the overall replication/reproducibility of results/experiments and other research outputs; visualization – preparation, creation and/or presentation of the published work, specifically visualization/data presentation; supervision – oversight and leadership responsibility for the research activity planning and execution, including mentorship external to the core team; writing – review & editing – preparation, creation and/or presentation of the published work by those from the original research group, specifically critical review, commentary, or revision – including pre‐ or post‐publication stages.

## Data Availability

The data that support the findings of this study are available on request from the corresponding author. The data are not publicly available as they contain information that could compromise the privacy of research participants.

## References

[1] Bundy C . Visible difference associated with disease: skin conditions. In: Rumsey N , Harcourt D , editors. The Oxford handbook of: the psychology of appearance. Oxford: Oxford University Press; 2012. p. 398–413

[2] Raychaudhuri SK , Maverakis E , Raychaudhuri SP . Diagnosis and classification of psoriasis. Autoimmun Rev. 2014;13:490–5. 10.1016/j.autrev.2014.01.008 24434359

[3] Shenoi SD . Psychophysiologic skin disorders. In: Latheef A , editor. Handbook of psychodermatology. New Delhi: Jaypee Brothers Medical Publishers Pvt Ltd; 2016. p. 63.

[4] Aydin E , Atis G , Bolu A , Aydin C , Karabacak E , Dogan B , et al. Identification of anger and self‐esteem in psoriasis patients in a consultation‐liaison psychiatry setting: a case control study. Psychiatry Clin Psychopharmacol. 2017;27:216–20. 10.1080/24750573.2017.1326740

[5] Martín‐Brufau R , Romero‐Brufau S , Martín‐Gorgojo A , Brufau Redondo C , Corbalan J , Ulnik J . Psoriasis lesions are associated with specific types of emotions. Emotional profile in psoriasis. Eur J Dermatol. 2015;25:329–34. 10.1684/ejd.2015.2577 26065886

[6] Kossakowska MM , Cieścińska C , Jaszewska J , Placek W . Control of negative emotions and its implication for illness perception among psoriasis and vitiligo patients. J Eur Acad Dermatol Venereol. 2010;24:429–33. 10.1111/j.1468-3083.2009.03432.x 19744257

[7] Crosta ML , De Simone C , Di Pietro S , Acanfora M , Caldarola G , Moccia L , et al. Childhood trauma and resilience in psoriatic patients: a preliminary report. J Psychosom Res. 2018;106:25–8. 10.1016/j.jpsychores.2018.01.002 29455895

[8] Devrimci‐Ozguven H , Kundakci N , Kumbasar H , Boyvat A . The depression, anxiety, life satisfaction and affective expression levels in psoriasis patients. J Eur Acad Dermatol Venereol. 2000;14:267–71. 10.1046/j.1468-3083.2000.00085.x 11204514

[9] Malhotra SK , Mehta V . Role of stressful life events in induction or exacerbation of psoriasis and chronic urticaria. Indian J Dermatol Venereol Leprol. 2008;74:594–9. 10.4103/0378-6323.45100 19171981

[10] Simonić E , Kaštelan M , Peternel S , Pernar M , Brajac I , Rončević‐gržeta I , et al. Childhood and adulthood traumatic experiences in patients with psoriasis. J Dermatol. 2010;37:793–800. 10.1111/j.1346-8138.2010.00870.x 20883363

[11] Erfanian M . Childhood trauma: a risk for major depression in patients with psoriasis. Psychiatry Clin Psychopharmacol. 2018;28:378–85. 10.1080/24750573.2018.1452521

[12] Rousset L , Halioua B . Stress and psoriasis. Int J Dermatol. 2018;57:1165–72. 10.1111/ijd.14032 29729012

[13] Dowlatshahi EA , Van Der Voort EAM , Arends LR , Nijsten T . Markers of systemic inflammation in psoriasis: a systematic review and meta‐analysis. Br J Dermatol. 2014;169:266–82. 10.1111/bjd.12355 23550658

[14] Papadopoulos L , Walker C . Understanding skin problems: acne, eczema, psoriasis and related conditions. Chichester: John Wiley & Sons Ltd; 2003.

[15] Jensen P , Ahlehoff O , Egeberg A , Gislason G , Hansen P , Skov L . Psoriasis and new‐onset depression: a Danish nationwide cohort study. Acta Derm Venereol. 2016;96:39–42. 10.2340/00015555-2183 26086213

[16] Thompson A , Kent G . Adjusting to disfigurement: processes involved in dealing with being visibly different. Clin Psychol Rev. 2001;21:663–82. 10.1016/S0272-7358(00)00056-8 11434225

[17] Bahmer JA , Kuhl J , Bahmer FA . How do personality systems interact in patients with psoriasis, atopic dermatitis and urticaria? Acta Derm Venereol. 2007;87:317–24. 10.2340/00015555-0246 17598034

[18] Hughes O , Hutchings PB , Phelps C . Stigma, social appearance anxiety and coping in men and women living with skin conditions: a mixed methods analysis. Skin Health Dis. 2021;e73. 10.1002/ski2.73 PMC9720193 36479270

[19] Solovan C , Marcu M , Chiticariu E . Life satisfaction and beliefs about self and the world in patients with psoriasis: a brief assessment. Eur J Dermatol. 2014;24:242–7. 10.1684/ejd.2014.2295 24721720

[20] Rumsey N , Harcourt D . Body image and disfigurement: issues and interventions. Body Image. 2004;1:83–97. 10.1016/S1740-1445(03)00005-6 18089143

[21] Picardi A , Mazzotti E , Gaetano P , Cattaruzza MS , Baliva G , Melchi CF , et al. Stress, social support, emotional regulation, and exacerbation of diffuse plaque psoriasis. Psychosomatics. 2005;46:556–64. 10.1176/appi.psy.46.6.556 16288135

[22] Sampogna F , Tabolli S , Abeni D , IDI Multipurpose Psoriasis Research on Vital Experiences (IMPROVE) investigators. Living with psoriasis: prevalence of shame, anger, worry, and problems in daily activities and social life. Acta Derm Venereol. 2012;92:299–303. 10.2340/00015555-1273 22678565

[23] Coneo AMC , Thompson AR , Lavda A , The Appearance Research Collaboration . The influence of optimism, social support, and anxiety on aggression in a sample of dermatology patients: an analysis of cross‐sectional data. Br J Dermatol. 2017;176:1187–94. 10.1111/bjd.15115 27726126

[24] Altinoz AE , Taskintuna N , Altinoz ST , Ceran S . A cohort study of the relationship between anger and chronic spontaneous urticaria. Adv Ther. 2014;31:1000–7. 10.1007/s12325-014-0152-6 25209876

[25] Conrad R , Geiser F , Haidl G , Hutmacher M , Liedtke R , Wermter F . Relationship between anger and pruritus perception in patients with chronic idiopathic urticaria and psoriasis. J Eur Acad Dermatol Venereol. 2008;22:1062–9. 10.1111/j.1468-3083.2008.02714.x 18355189

[26] Matussek P , Agerer D , Seibt G . Aggression in depressives and psoriatics. Psychother Psychosom. 1985;43:120–5. 10.1159/000287868 4001298

[27] Koo J , Lebwohl A . Psychodermatology: the mind and skin connection. Am Fam Physician. 2001;64:1873–8. 11764865

[28] Fried RG , Friedman S , Paradis C , Hatch M , Lynfield Y , Duncanson C , et al. Trivial or terrible? The psychosocial impact of psoriasis. Int J Dermatol. 1995;34:101–5. 10.1111/j.1365-4362.1995.tb03588.x 7737765

[29] Prussick L , Jimenez E , Nussbaum D , Prussick R . Psoriasis and psychological comorbidities. J Psoriasis Psoriatic Arthritis. 2016;1:80–5. 10.1177/247553031600100206

[30] Shah RB . Impact of collaboration between psychologists and dermatologists: UK hospital system example. Int J Womens Dermatol. 2018;4:8–11. 10.1016/j.ijwd.2017.10.003 29872670 PMC5986107

[31] Mental health and skin disease. London: All‐Party Parliamentary Group on Skin; 2020. Available from: http://www.appgs.co.uk/wp‐content/uploads/2020/09/Mental_Health_and_Skin_Disease2020.pdf

[32] Hughes O , Hunter R . The importance of exploring the role of anger in people with psoriasis. JMIR Dermatol. 2022. 10.2196/33920 PMC10334900 37632869

[33] Hunter R , Lewis S , Noble S , Rance J , Bennett PD . Post‐thrombotic panic syndrome”: a thematic analysis of the experience of venous thromboembolism. Br J Health Psychol. 2017;22:8–25. 10.1111/bjhp.12213 27611117

[34] Hunter R , Parry B , Thomas C . Fears for the future: a qualitative exploration of the experiences of individuals living with multiple sclerosis, and its impact upon the family from the perspective of the person with MS. Br J Health Psychol. 2020;26:464–81. 10.1111/bjhp.12495 33340208

[35] Martin C , Bonas S , Shepherd L , Hedges E . The experience of scar management for adults with burns: an interpretative phenomenological analysis. Burns. 2016;42:1311–22. 10.1016/j.burns.2016.03.002 27033802

[36] Egan K , Harcourt D , Rumsey N , The Appearance Research Collaboration . A qualitative study of the experiences of people who identify themselves as having adjusted positively to a visible difference. J Health Psychol. 2011;16:739–49. 10.1177/1359105310390246 21346018

[37] Sharratt ND , Jenkinson E , Moss T , Clarke A , Rumsey N . Understandings and experiences of visible difference and romantic relationships: a qualitative exploration. Body Image. 2018;27:32–42. 10.1016/j.bodyim.2018.08.002 30125758

[38] Trier‐Bieniek A . Framing the telephone interview as a participant‐centred tool for qualitative research: a methodological discussion. Qual Res. 2012;12:630–44. 10.1177/1468794112439005

[39] Keary E , Hevey D , Tobin AM . A qualitative analysis of psychological distress in hidradenitis suppurativa. Br J Dermatol. 2020;182:342–7. 10.1111/bjd.18135 31099891

[40] Braun V , Clarke V . To saturate or not to saturate? Questioning data saturation as a useful concept for thematic analysis and sample‐size rationales. Qual Res in Sport Exerc Health. 2021;13(2):201–16. 10.1080/2159676X.2019.1704846

[41] Braun V , Clarke V . Conceptual and design thinking for thematic analysis. Qual Psychol. 2021. 10.1037/qup0000196

[42] Saunders B , Sim J , Kingstone T , Baker S , Waterfield J , Bartlam B , et al. Saturation in qualitative research: exploring its conceptualization and operationalization. Qual Quantity. 2018;52:1893–907. 10.1007/s11135-017-0574-8 PMC5993836 29937585

[43] O’Reilly M , Parker N . ‘Unsatisfactory saturation’: a critical exploration of the notion of saturated sample sizes in qualitative research. Qual Res. 2013;13:190–7. 10.1177/1468794112446106

[44] Malterud K , Siersma VD , Guassora AD . Sample size in qualitative interview studies: guided by information power. Qual Health Res. 2016;26(13):1753–60. 10.1177/1049732315617444 26613970

[45] Braun V , Clarke V . Using thematic analysis in psychology. Qual Res Psychol. 2006;3:77–101. 10.1191/1478088706qp063oa

[46] Wu Y , Mills D , Bala M . Impact of psoriasis on patients’ work and productivity. Am J Clin Dermatol. 2009;10:407–10. 10.2165/11310440-000000000-00000 19824741

[47] Roberts AL , Malspeis S , Kubzansky LD , Feldman CH , Chang S‐C , Koenen KC , et al. Association of trauma and posttraumatic stress disorder with incident systemic lupus erythematosus in a longitudinal cohort of women. Arthritis Rheumatol. 2017;69:2162–9. 10.1002/art.40222 28929625 PMC5659907

[48] Kimyai‐Asadi A , Usman A . The role of psychological stress in skin disease. J Cutan Med Surg. 2001;5:140–5. 10.1007/BF02737869 11443487

[49] Kirby B , Richards HL , Mason DL , Fortune DG , Main CJ , Griffiths CEM . Alcohol consumption and psychological distress in patients with psoriasis. Br J Dermatol. 2008;158:138–40. 10.1111/j.1365-2133.2007.08299.x 17999698

[50] Ferrão YA , Shavitt RG , Prado H , Fontenelle LF , Malavazzi DM , de Mathis MA , et al. Sensory phenomena associated with repetitive behaviors in obsessive‐compulsive disorder: an exploratory study of 1001 patients. Psychiatry Res. 2012;197:253–8. 10.1016/j.psychres.2011.09.017 22361443

[51] Ben‐Sasson A , Podoly TY . Sensory over responsivity and obsessive‐compulsive symptoms: a cluster analysis. Compr Psychiatry. 2017;73:151–9. 10.1016/j.comppsych.2016.10.013 27978503

[52] Jafferany M , Afrin A , Mkhoyan R , Khemani U , Sadoughifar R . Therapeutic implications of personality disorders in dermatology. Dermatol Ther. 2020;33:1–5. 10.1111/dth.13910 32594602

[53] Hajcak G , Franklin ME , Simons RF , Keuthen NJ . Hairpulling and skin picking in relation to affective distress and obsessive‐compulsive symptoms. J Psychopathol Behav Assess. 2006;28:177–85. 10.1007/s10862-005-9001-x

[54] Mavrogiorgou P , Bader A , Stockfleth E , Juckel G . Obsessive‐compulsive disorder in dermatology. J Dtsch Dermatol Ges. 2015;13:991–9. 10.1111/ddg.12781 26408459

[55] Hern S , Stanton AWB , Mellor R , Mortimer PS , Levick JR . Control of cutaneous blood vessels in psoriatic plaques. J Investig Dermatol. 1999;113:127–32. 10.1046/j.1523-1747.1999.00638.x 10417631

[56] Ismail E , Capo A , Amerio P , Merla A . Functional‐thermoregulatory model for the differential diagnosis of psoriatic arthritis. Biomed Eng Online. 2014;13:162. 10.1186/1475-925X-13-162 25494626 PMC4320504

[57] Sakson‐Obada O , Pawlaczyk M , Gerke K , Adamski Z . Acceptance of psoriasis in the context of body image, body experience, and social support. Health Psychol Rep. 2017;5:251–7. 10.5114/hpr.2017.63824

[58] Fortune DG , Richards HL , Main CJ , Griffiths CEM . Patients' strategies for coping with psoriasis. Clin Exp Dermatol. 2002;27:177–84. 10.1046/j.1365-2230.2002.01055.x 12072002

[59] Vachatova S , Andrys C , Krejsek J , Salavec M , Ettler K , Rehacek V , et al. Metabolic syndrome and selective inflammatory markers in psoriatic patients. J Immunol Res. 2016;2016:1–8. 10.1155/2016/5380792 PMC5209622 28097156

[60] Sokolova MV , Simon D , Nas K , Zaiss MM , Luo Y , Zhao Y , et al. A set of serum markers detecting systemic inflammation in psoriatic skin, entheseal, and joint disease in the absence of C‐reactive protein and its link to clinical disease manifestations. Arthritis Res Ther. 2020;22:1–8. 10.1186/s13075-020-2111-8 32051028 PMC7017480

[61] Remröd C , Sjöström K , Svensson Å . Psychological differences between early‐and late‐onset psoriasis: a study of personality traits, anxiety and depression in psoriasis. Br J Dermatol. 2013;169:344–50. 10.1111/bjd.12371 23565588

